# The envelope proteins from SARS-CoV-2 and SARS-CoV potently reduce the infectivity of human immunodeficiency virus type 1 (HIV-1)

**DOI:** 10.1186/s12977-022-00611-6

**Published:** 2022-11-19

**Authors:** Wyatt Henke, Hope Waisner, Sachith Polpitiya Arachchige, Maria Kalamvoki, Edward Stephens

**Affiliations:** Department of Microbiology, Molecular Genetics and ImmunologyUniversity of Kansas Medical Center, 2000 Hixon Hall 3901 Rainbow Blvd, Kansas, KS 66160 USA

## Abstract

**Background:**

Viroporins are virally encoded ion channels involved in virus assembly and release. Human immunodeficiency virus type 1 (HIV-1) and influenza A virus encode for viroporins. The human coronavirus SARS-CoV-2 encodes for at least two viroporins, a small 75 amino acid transmembrane protein known as the envelope (E) protein and a larger 275 amino acid protein known as Orf3a. Here, we compared the replication of HIV-1 in the presence of four different β-coronavirus E proteins.

**Results:**

We observed that the SARS-CoV-2 and SARS-CoV E proteins reduced the release of infectious HIV-1 yields by approximately 100-fold while MERS-CoV or HCoV-OC43 E proteins restricted HIV-1 infectivity to a lesser extent. Mechanistically, neither reverse transcription nor mRNA synthesis was involved in the restriction. We also show that all four E proteins caused phosphorylation of eIF2-α at similar levels and that lipidation of LC3-I could not account for the differences in restriction. However, the level of caspase 3 activity in transfected cells correlated with HIV-1 restriction in cells. Finally, we show that unlike the Vpu protein of HIV-1, the four E proteins did not significantly down-regulate bone marrow stromal cell antigen 2 (BST-2).

**Conclusions:**

The results of this study indicate that while viroporins from homologous viruses can enhance virus release, we show that a viroporin from a heterologous virus can suppress HIV-1 protein synthesis and release of infectious virus.

**Supplementary Information:**

The online version contains supplementary material available at 10.1186/s12977-022-00611-6.

## Background

The recent introduction of the highly transmissible coronavirus SARS-CoV-2 into the human population has resulted in the COVID-19 respiratory disease and pandemic [1–4]. This has highlighted the urgent need to better understand the function of viral proteins in replication and virus pathogenesis. The human pathogenic β-coronaviruses (SARS-CoV-2, SARS-CoV, MERS-CoV, HCoV-OC43) contain four structural proteins (S, M, E, and N) [5, 6]. The spike (S) protein, the largest protein within virions, binds to the angiotensin-converting enzyme 2 (ACE-2) receptor and is the major target for neutralizing antibodies. The membrane (M) protein, the most abundant protein of the virion, is thought to form a scaffold in the ER-Golgi intermediate compartment (ERGIC), the site of virus maturation [7, 8]. The nucleocapsid (N) of SARS-CoV binds to the viral RNA and together with E and M can form viral particles [9].

The E protein of the β-coronaviruses is the smallest and least abundant virion protein. This transmembrane protein (75–84 amino acids in length) is predicted to have a short N-terminal domain, a hydrophobic transmembrane domain, and a longer cytoplasmic domain. Recent studies indicate that the E protein spans the membrane a single time with the N-terminal domain facing the lumen of the ER/ERGIC/Golgi [10–12]. While the expression of the SARS-CoV E protein is dispensable for coronavirus replication, its deletion results in reduced virus growth, likely due to inefficient assembly [13–15]. The E proteins of SARS-CoV-2 and SARS-CoV have approximately 95% amino acid identity, differing at only four amino acid positions, while E proteins of MERS-CoV and HCoV-OC43 are more distantly related to SARS-CoV-2 E protein with approximately 37% and 25% amino acid identity, respectively. Interestingly, the E proteins of SARS-CoV-2 and several bat coronavirus isolates (RATG13, ZC45, and ZXC21) are identical.

The E proteins of coronaviruses have similarities to the Vpu protein of HIV-1 concerning its size, orientation in the membrane, ion channel activity, and functions to enhance virus release [16–23]. These similarities prompted us to examine the biological properties of the SARS-CoV-2 E protein in the context of HIV-1 to determine if the E protein could substitute for the Vpu protein of HIV-1. Thus, the purpose of the study was to better understand the properties of this viroporin. Our results indicate that the SARS-CoV-2 and SARS-CoV E proteins potently restricted both HIV-1 and HIV-1Δ*vpu* infections. This restriction was not due to inhibition of viral integration, synthesis of viral RNA, phosphorylation of eIF2-α from ER stress, or activation of autophagy but correlated with caspase 3 activity. These results show for the first time the E viroporin from SARS-CoV-2 that enhances virus infection can restrict a heterologous virus (i.e., HIV-1).

## Results

### The E protein of SARS-CoV-2 is expressed within the RER, ERGIC, and Golgi complex and potently restricts the release of infectious HIV-1

**Fig. 1:**
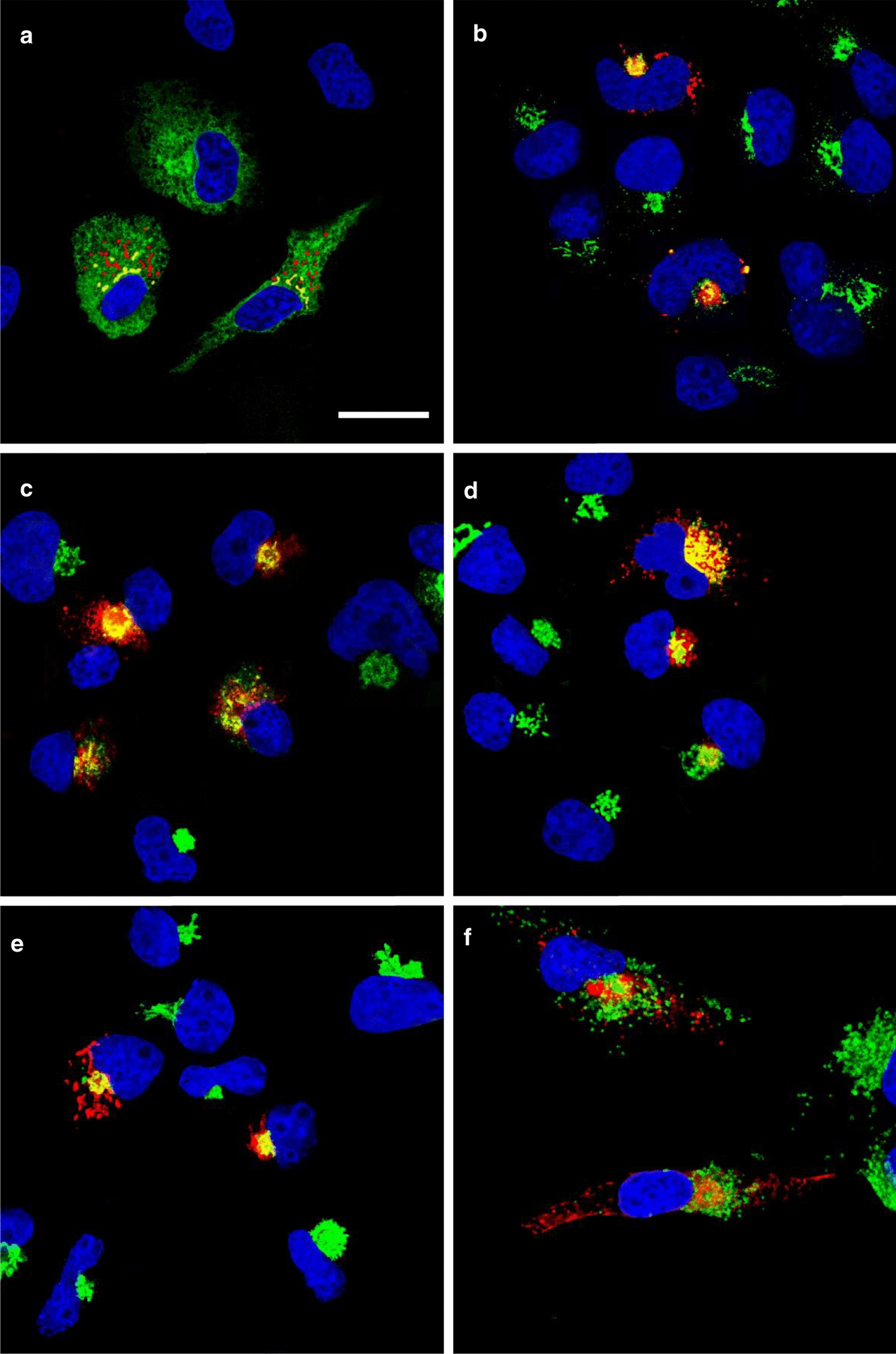
The expression of the SARS-CoV-2 E protein is restricted to intracellular compartments of the cell. COS-7 cells were transfected with the vector expressing the SARS-CoV-2 E-HA protein. At 24 h, cells were processed for immunostaining. The cells on coverslips were reacted with a mouse monoclonal antibody against HA-tag and with rabbit antibodies against ERGIC53, Golgin-97, or LAMP-1. The cells were washed and reacted with a secondary goat anti-rabbit antibody conjugated to AlexaFluor488 and with a chicken anti-mouse antibody conjugated to AlexaFluor594 for 1 h. Cells on coverslips were counterstained with DAPI and mounted on glass slides with a glycerol-containing mounting medium. To examine the intracellular localization of the E protein with the RER, cis-medial Golgi, or *trans* Golgi network (TGN), COS-7 cells were co-transfected with the plasmid expressing the E-HA protein, and vectors expressing ERMoxGFP, mNeonGreen-Giantin, or TGN38-GFP. Cells were prepared as above and immunostained with an anti-HA antibody and mounted as above. Coverslips were viewed with a Leica TCS SP8 Confocal Microscope with a 100X objective and a 2X digital zoom using the Leica Application Suite X (LASX) as previously described. A 405 nm filter was used to visualize the DAPI, a 594 nm filter to visualize HA staining, and a 488 nm filter to visualize the cellular markers. **A**. Cells co-transfected with vectors expressing SARS-CoV-2 E-HA and ERMoxGFP and immunostained with an anti-HA antibody. Scale bar = 20 µm. **B**. Cells transfected with a vector expressing SARS-CoV-2 E-HA and immunostained with anti-HA and anti-ERGIC53. **C**. Cells co-transfected with vectors expressing SARS-CoV-2 E-HA and mNeonGreen-Giantin and immunostained with an anti-HA antibody. **D**. Cells transfected with a vector expressing SARS-CoV-2 E-HA and immunostained with anti-HA and anti-Golgin97. **E** Cells co-transfected with vectors expressing SARS-CoV-2 E-HA and TGN38-GFP and immunostained with an anti-HA antibody. **F** Cells transfected with the vector expressing SARS-CoV-2 E-HA and immunostained with an anti-HA and anti-LAMP1

**Fig. 2:**
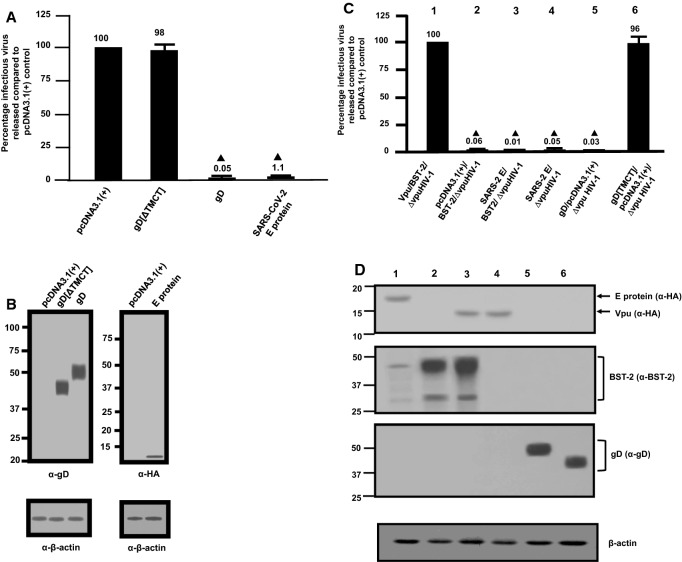
Release of infectious HIV-1Δ*vpu* and HIV-1 in the presence of SARS-CoV- 2 E protein. HEK293 cells were co-transfected with either the empty pcDNA3.1( +) vector or vectors expressing the HSV-1 gD[ΔTMCT], HSV-1 gD, or SARS-CoV-2 E protein and pNL4-3. At 48 h, the culture supernatants were collected and subjected to low-speed centrifugation to remove cellular debris, and levels of infectious virus released into the culture supernatants were determined using TZM-bl cell assays. **A** The level of HIV-1 infectivity in the culture medium from cells co-transfected with either the empty pcDNA3.1( +) vector or vectors expressing gD[ΔTMCT], gD, or SARS-CoV-2 E protein and pNL4-3. **B** Expression of the gD proteins or E protein from restriction assays in (**A**). Expression of the E protein or gD proteins from restriction assays in (B) was normalized with β-actin using an anti-β-actin antibody and immunoblots. **C** HEK293 cells were co-transfected with either the empty pcDNA3.1( +) vector or vectors expressing 1) Vpu/BST-2/∆*vpu*HIV-1; 2) pcDNA3.1( +)/BST-2**/**∆*vpu*HIV-1; 3) SARS-CoV- 2 E/BST-2//∆*vpu*HIV-1; 4) SARS-CoV-2 E/∆*vpu*HIV-1; 5) gD/pcDNA3.1( +)/∆*vpu*HIV-1; or 6) gD[TMCT]/pcDNA3.1( +)/∆*vpu*HIV-1. At 48 h, culture supernatants were collected as described above and assayed for infectious HIV-1. The numbers above the figure correspond to the lanes in Panel D. **D** The cells collected from the experiments were lysed. Lysates were normalized for β-actin and analyzed for Vpu or E proteins using an anti-HA antibody (upper panel); BST-2 using an anti-BST-2 antibody (middle panel); gD proteins using an anti-gD monoclonal antibody (lower panel). The blot for β-actin is shown at the bottom. All restriction assays in Panels A and C were performed at least four times and statistical differences from the pcDNA3.1( +)/HIV-1 control was evaluated using a two-tailed Students *t*-test, with *p* < 0.01 () considered significant

We first examined the intracellular expression of the SARS-CoV-2 E protein. Previous studies have shown that the E protein predominantly localizes to the ERGIC and Golgi complex in SARS-CoV infected cells although this protein is synthesized on the rough endoplasmic reticulum (RER) [12]. COS-7 cells were transfected with the vector expressing the SARS-CoV-2 E protein tagged at the C-terminus with an HA-tag and immunostained for the presence of the E protein (anti-HA) and with antibodies directed against ERGIC53 (ER-Golgi intermediate compartment; Fig. 1b), Golgin97 (*trans* Golgi; Fig. 1d), or LAMP-1 (late endosomes/lysosomes; Fig. 1f). To analyze the co-localization of the E protein with the endoplasmic reticulum, cis-medial Golgi, and *trans* Golgi network (TGN), cells were co-transfected with the plasmid expressing the E-HA protein and vectors expressing ER-MoxGFP (RER: Fig. 1a), giantin-GFP (cis/medial Golgi: Fig. 1c), or TGN38-GFP (TGN: Fig. 1e). In these co-transfections, cells were fixed, permeabilized, and immunostained for E protein using an anti-HA antibody as described in the Materials and Methods section. The results show that the E protein co-localized with markers for the ER, ERGIC, cis-medial Golgi, trans Golgi, and TGN (Fig. 1). However, the E protein was neither observed on the cell plasma membrane nor in the lysosomes (as indicated by staining with LAMP-1) (Fig. 1f) [11, 24–28]. We also compared the intracellular localization of SARS-CoV-2 E with the E proteins of SARS-CoV, MERS-CoV, and HcoV-OC43 (Additional files 1, 2 and 3). Our results indicate that the intracellular localization of these three E proteins was similar to the SARS-CoV-2 E protein. We next determined if the SARS-CoV-2 E protein would enhance or decrease the level of infectious HIV-1 or ∆*vpu*HIV-1 produced. HEK293 cells were co-transfected with the empty pcDNA3.1( +) vector, or pcDNA3.1( +) expressing either the SARS-CoV-2 E protein, HSV-1 gD (positive control for restriction), or gD[ΔTMCT] (a truncated form of gD that is secreted from cells and does not restrict HIV-1) and pNL4-3 [29, 30]. Our results indicated that the level of infectious HIV-1 released was significantly decreased in the presence of the SARS-CoV-2 E protein (Fig. 2a). Immunoblots for the gD and SARS-CoV-2 E proteins (anti-HA for the E protein; anti-gD for HSV-1 gD) from the cell lysates revealed that these proteins were expressed well during the co-transfections (Fig. 2b). We also examined whether the E protein would restrict ∆*vpu*HIV-1. The protein BST-2 is known to reduce the amount of HIV-1 in the absence of the Vpu protein. However, BST-2 is not expressed in HEK293 cells. To determine if the SARS-CoV-2 E protein could substitute for the Vpu, we examined the ability of ∆*vpu*HIV-1 to replicate in the presence of BST-2 and SARS-CoV-2 E protein. HEK293 cells were co-transfected with various combinations of plasmids expressing: (a) Vpu/BST-2/Δ*vpu*HIV-1; (b) empty pcDNA3.1( +)/BST-2/Δ*vpu*HIV-1; (c) SARS-CoV-2-E/BST-2/Δ*vpu*HIV-1; (d) pcDNA3.1( +)/SARS-CoV-2 E/Δ*vpu*HIV-1; (e) pcDNA3.1( +)/gD/Δ*vpu*HIV-1; or (f) pcDNA3.1( +)/gD[ΔTMCT]/Δ*vpu*HIV-1. (Fig. 2c). Our results indicated that co-transfection with vectors expressing SARS-CoV-2 E protein, BST-2, and the ∆*vpu*HIV-1 genome also resulted in decreased production of infectious virus, although not as great as the gD protein of HSV-1 (Fig. 2c). Immunoblots for gD, the SARS-CoV-2 E protein, Vpu and BST-2 (anti-HA for the E protein and Vpu; anti-gD for HSV-1 gD; anti-BST -2) from the cell lysates also revealed that these proteins were expressed well during the co-transfections (Fig. 2b). Taken together, these results indicate that the SARS-CoV-2 E protein inhibited the production of both viruses and that the E protein could not substitute for the Vpu protein.

### The SARS-CoV-2 E protein did not inhibit HSV-1 infection

**Fig. 3:**
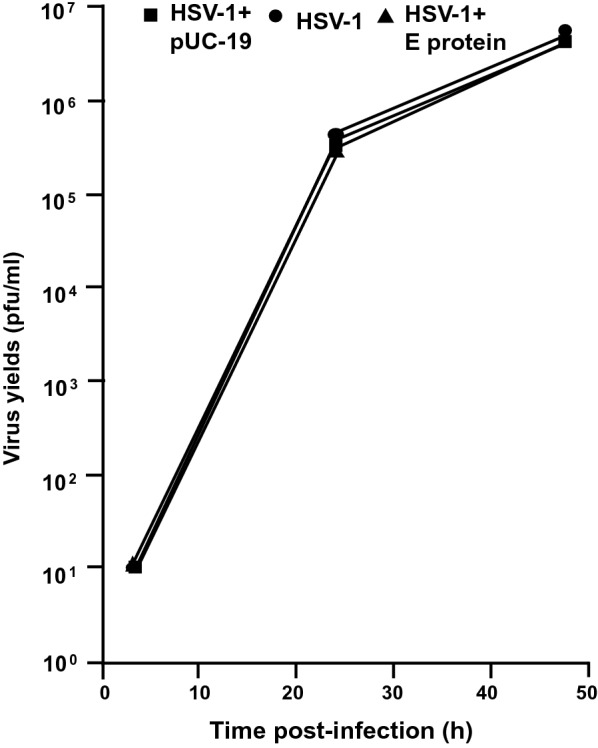
The E protein of SARS-CoV-2 does not restrict the replication of HSV-1. HEK293 cells were left untransfected or transfected with the empty vector (pUC-19) or a vector expressing the SARS-CoV-2 E protein. At 24 h post-transfection, cells were inoculated with HSV-1 (0.01 pfu/cell). Cells were harvested at 24 and 48 h post-infection. The number of plaque-forming units was determined using standard plaque assays

We next determined if E protein could decrease the production of infectious HSV-1 virus. HEK293 cells were transfected with the plasmid expressing SARS-CoV-2 E protein for 24 h followed by infection of cultures with HSV-1 (0.01 pfu/cell). Samples were collected at 3, 24, and 48 h post-infection. The results indicate that SARS-CoV-2 E protein did not interfere with infectious HSV-1 progeny virus production at 3, 24, or 48 h post-infection (Fig. 3). This is not surprising as HSV-1 expresses proteins known as γ_1_34.5 which is known to inhibit the phosphorylation of eIF2-α, and Us3, known to inhibit apoptosis and autophagy [31–34].

### Restriction of HIV-1 by SARS-CoV, MERS-CoV, and HCoV-OC43 E proteins

**Fig. 4:**
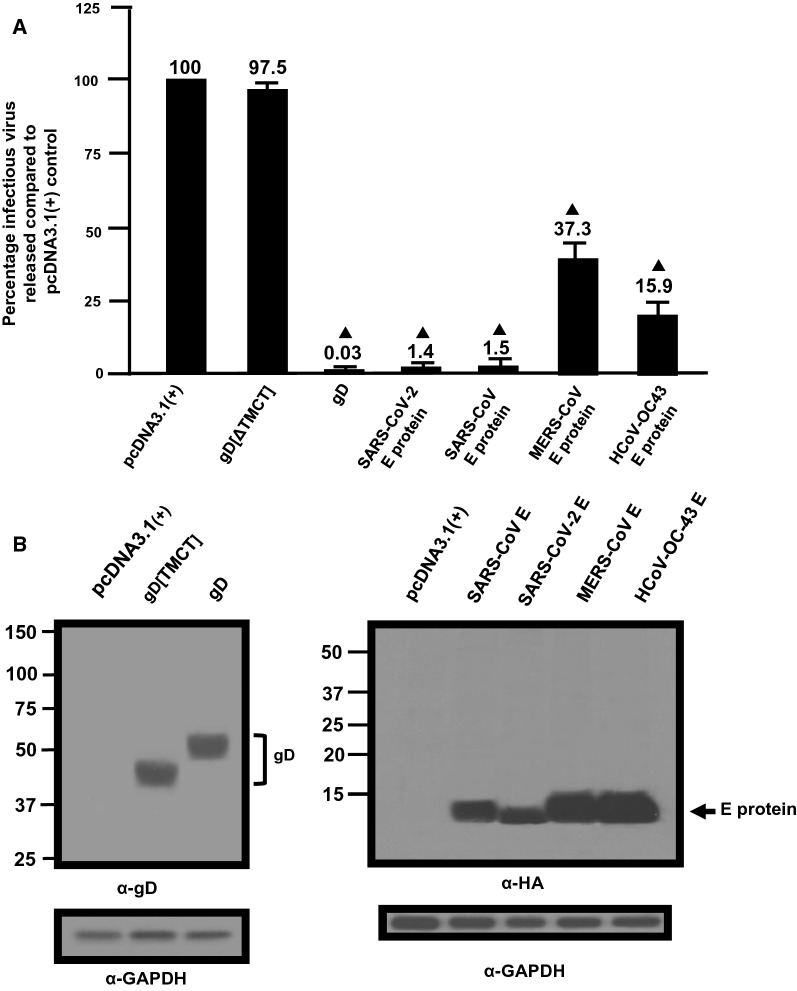
Restriction of infectious HIV-1 production by the SARS-CoV, MERS-CoV, and HCoV-OC43 E proteins. **A** The level of infectious HIV-1 released into the culture medium from cells co-transfected with pcDNA3.1( +) alone, or vectors expressing SARS-CoV-2 E-HA, SARS-CoV E-HA, MERS-CoV E-HA, HCoV-OC43 E-HA, HSV-1 gD, or gD[ΔTMCT] and pNL4-3. **B** Immunoprecipitation of gD and gD[ΔTMCT] from the cell lysates of the restriction assay in Panel A using an anti-gD antibody. **C** Immunoprecipitation of the various E proteins from the cell lysates from the restriction assay in Panel A using an anti-HA antibody

As the E protein of SARS-CoV-2 appeared to decrease the levels of HIV-1 virus released, we analyzed pcDNA3.1( +) vectors that expressed the E proteins from three other β-coronaviruses that cause mild to severe pathogenicity in humans (HCoV-OC43, SARS-CoV, MERS-CoV). Like the SARS-CoV-2 E protein, examination of the intracellular expression revealed that these E proteins were primarily localized in the ER and Golgi regions of the cell with no expression at the cell surface (Additional files 1, 2 and 3). We determined if these E proteins could also restrict HIV-1 particle infectivity by transfection of HEK293 cells with vectors expressing the SARS-CoV-2 E-HA, SARS-CoV E-HA, MERS-CoV E-HA, HCoV-OC43 E-HA, HSV-1 gD, or gD[ΔTMCT] and pNL4-3. At 48 h, the culture medium was collected, clarified, and the levels of infectious HIV-1 released were determined using TZM.bl cell assays. The presence of gD restricted the release of infectious HIV-1 (0.04%) while the presence of gD [TMCT] did not affect levels of infectious virus released (~ 101%) (Fig. 4a). Our results indicate that the E proteins from SARS-CoV-2 and SARS-CoV potently restricted the release of infectious HIV-1 at 1.3% and 1.4%, respectively. This is not surprising as these two proteins have ~ 95% amino acid identity. However, MERS-CoV and HCoV-OC43 E proteins were less restrictive at approximately 37% and 16%, respectively, of the pcDNA3.1( +) control (Fig. 4). Comparison of the amino acid sequences revealed that SARS-CoV-2 and MERS-CoV E proteins had ~ 37% amino acid identity while SARS-CoV-2 and HCoV-OC43 E proteins had ~ 26% amino acid identity. Immunoprecipitation of gD and E proteins from cell lysates from the restriction assays confirmed that the gD and E proteins were expressed (Fig. 4b). These results provide additional data on the specificity of the restriction of HIV-1.

### The SARS-CoV-2 and SARS-CoV E proteins result in a decreased biosynthesis of HIV-1 proteins

**Fig. 5:**
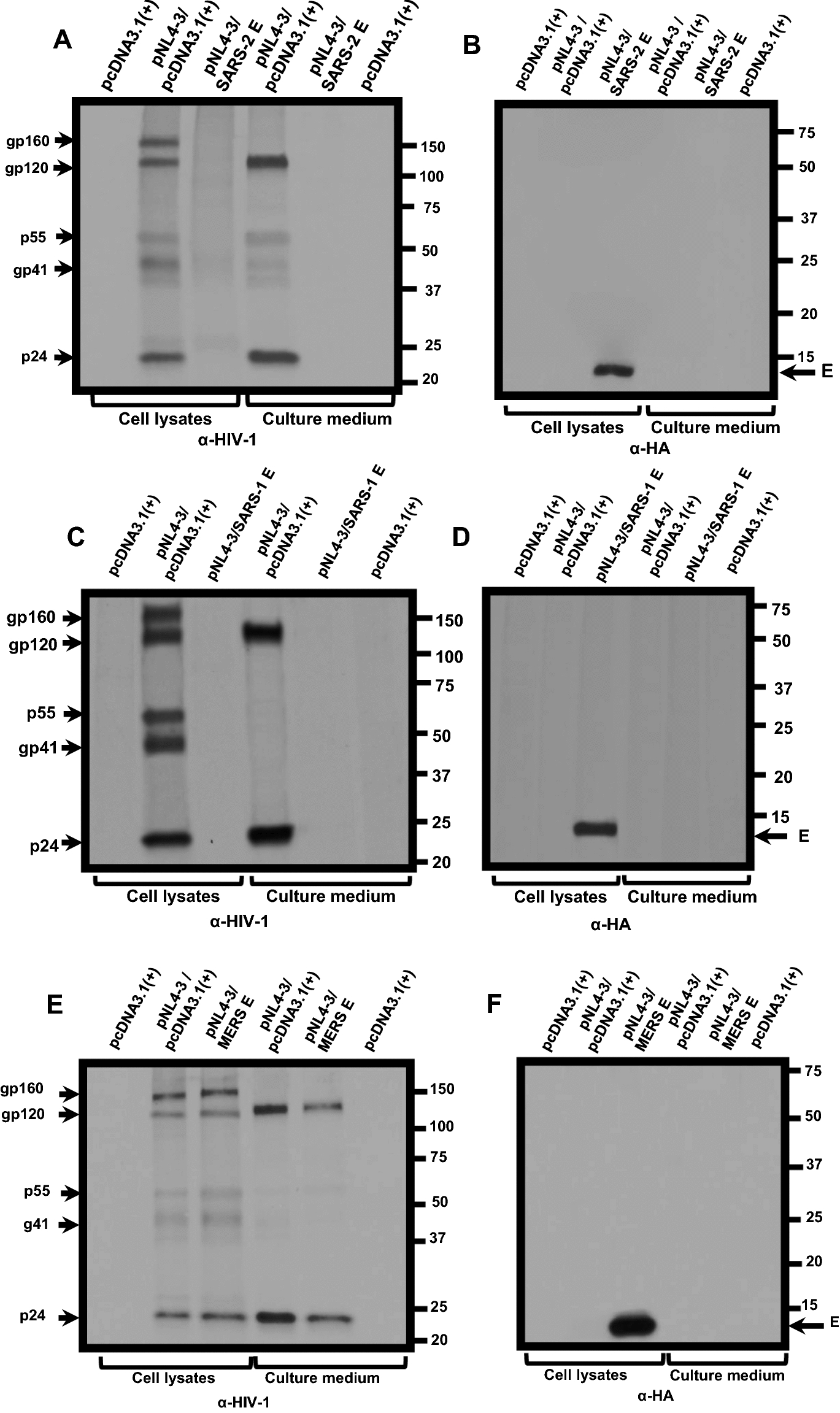

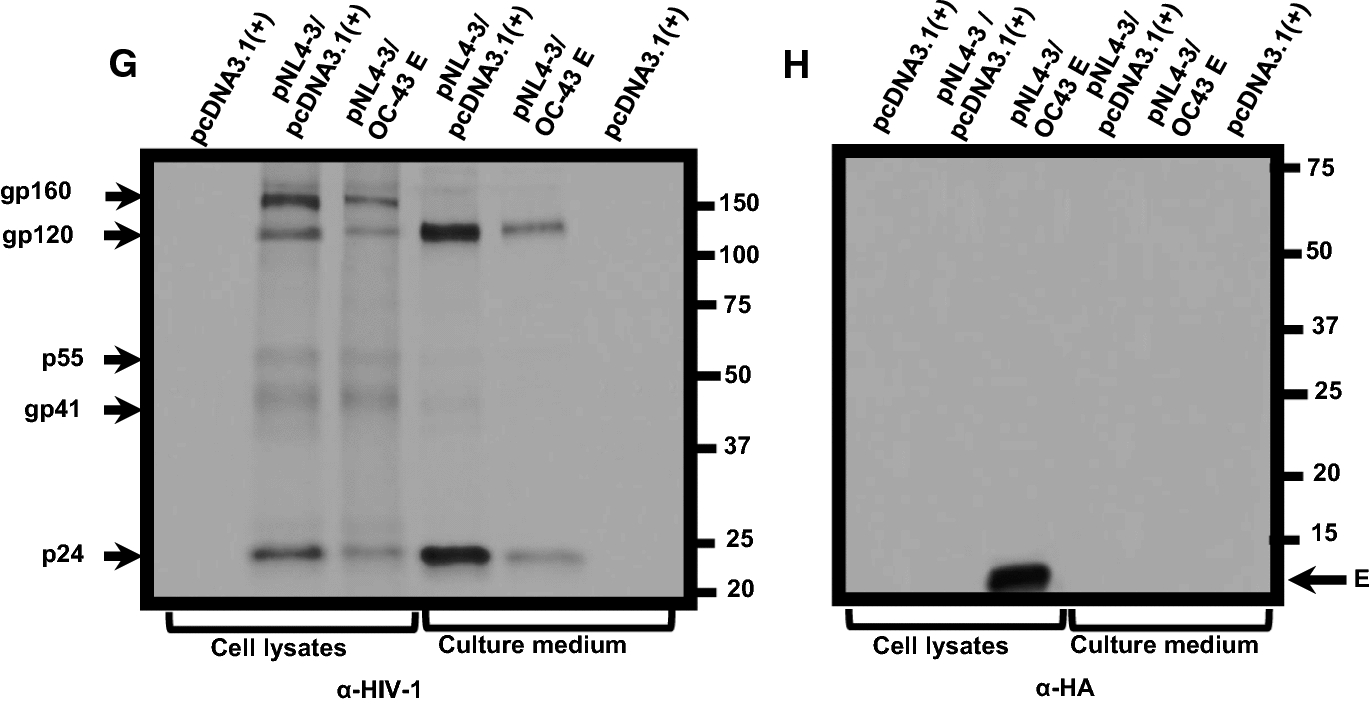
The biosynthesis of HIV-1 proteins is attenuated in the presence of β-coronavirus E proteins. HEK293 cells were transfected with either the empty pcDNA3.1( +) or vectors expressing SARS-CoV-2, SARS-CoV, MERS-CoV, or HCoV-OC43 E proteins and pNL4-3. At 30 h post-transfection, cells were starved of methionine/cysteine for 2 h and radiolabeled with ^35^S-methionine/cysteine for 16 h. The culture medium was collected, and cell lysates were prepared as described in the Materials and Methods section. HIV-1 proteins were immunoprecipitated using anti-HIV-1 antibodies while the E proteins were immunoprecipitated with antibodies directed against the HA-tag. The immunoprecipitates were collected on protein-A-Sepharose, washed, and boiled in sample-reducing buffer. The proteins were separated by SDS-PAGE and visualized using standard radiographic techniques. **A-B** Immunoprecipitation of HIV-1 and E proteins from cell lysates and culture medium from HEK293 cells co-transfected with either pcDNA3.1( +) alone, pcDNA3.1( +)/pNL4-3, or pcDNA3.1( +) expressing SARS-CoV-2 E-HA protein/pNL4-3. **C-D** Immunoprecipitation of HIV-1 and E proteins from cell lysates and culture medium from HEK293 cells co-transfected with pcDNA3.1( +) alone, or pcDNA3.1( +)/pNL4-3, or pcDNA3.1( +) expressing the SARS-CoV E-HA/pNL4-3. **E–F** Immunoprecipitation of HIV-1 and E proteins from cell lysates and culture medium from HEK293 cells co-transfected pcDNA3.1( +) alone, pcDNA3.1( +)/pNL4-3, or pcDNA3.1( +) expressing the MERS-CoV E-HA/pNL4-3. **G-H** Immunoprecipitation of HIV-1 and E proteins from cell lysates and culture medium from HEK2933 cells co-transfected pcDNA3.1( +) alone, pcDNA3.1( +)/pNL4-3, or pcDNA3.1( +) expressing the HCoV-OC43 E-HA/pNL4-3

We examined the mechanism through which the SARS-CoV-2 E protein restricted the release of infectious HIV-1. HEK293 cells were transfected with the empty pcDNA3.1( +) vector or one expressing the SARS-CoV-2 E protein and pNL4-3. At 36 h post-transfection, cells were starved for methionine/cysteine for 2 h and radiolabeled with ^35^S-methionine/cysteine for 16 h. The culture medium was collected and clarified while cell lysates were prepared. HIV-1 and E proteins were immunoprecipitated as described in the Materials and Methods section. Immunoprecipitation of HIV-1 proteins from cell lysates of cells co-transfected with pcDNA3.1( +) and pNL4-3 revealed the presence of the gp160 Env, gp120 cleavage product, Gag p55, and p24. Further, gp120 and p24 were immunoprecipitated from the clarified culture medium. In contrast, immunoprecipitation of HIV-1 proteins from lysates prepared from cells co-transfected with the vector expressing SARS-CoV-2 E and pNL4-3 revealed that the Env precursor gp160, the gp120, p55, and p24 were immunoprecipitated at much lower levels as were gp120 and p24 in the clarified culture medium (Fig. 5a). The E protein was detected in the cell lysates but not detected in the culture medium (Fig. 5b). We also determined if the E proteins from SARS-CoV, MERS-CoV, and HCoV-OC43 also affected HIV-1 protein synthesis (Fig. 5c–h). The presence of SARS-CoV E also reduced levels of viral proteins in the cell lysates and culture medium compared with the pcDNA3.1( +)/pNL4-3 control (Fig. 5c–d) while analysis of MERS-CoV E/pNL4-3 and HCoV-OC43 E/ pNL4-3 co-transfections revealed an intermediate level of viral proteins in the cell lysates and in the culture medium (Fig. 5e–h), which correlates with the levels of HIV-1 infectivity.

### The E protein does not prevent the integration of the HIV-1 genome or viral transcription

As the immunoprecipitation assays indicated that HIV-1 protein synthesis was decreased in the presence of the E protein, we examined the possible mechanism(s) that might be involved. Initially, we examined integration and viral RNA transcription. We used Alu-*gag* real-time PCR assays to quantify the integration of the HIV-1 genome in the presence and absence of the E protein. HEK293 cells were transfected with either the empty pcDNA3.1( +) vector or the vector expressing E-HA. At 24 h, equal infectious units of HIV-1 ΔEnv/VSV-G pseudotyped virus were used to infect the transfected HEK293 cells for 48 h. The DNA was extracted, and the levels of Alu-*gag* products were determined using the procedures in the Material and Methods section. Our results indicate that integration did not appear to be affected in the presence of the E protein while treatment of cells with raltegravir was effective in preventing integration (Additional file 4). We also quantified *tat* gene transcription by real-time RT-PCR and the results show that the number of transcripts detected during infection was not decreased in cells transfected with the vector expressing the SARS-CoV-2 E protein compared with the empty pcDNA3.1( +) vector (Additional file 4).

### Neither phosphorylation of eIF2-α nor induction of autophagy explains the restriction of HIV-1

As the expression of the SARS-CoV-2 E protein significantly reduced HIV-1 protein expression, we determined if the E protein activated pathways associated with ER stress and an unfolded protein response such as phosphorylation of eIF2-α, which can result in a generalized restriction of protein synthesis. HEK293 cells were transfected with the vector expressing the SARS-CoV-2 E-HA. At 48 h post-transfection (pt) cells were lysed and phosphorylation of eIF2-α was analyzed by immunoblots using an antibody against phosphorylated eIF2-α. In cells transfected with the empty pUC19 vector, little phosphorylated eIF2-α was detected (Additional file 5). In contrast, cells transfected with the vector expressing the SARS-CoV-2 E-HA resulted in the phosphorylation of eIF2-α (Additional file 5). To determine if the eIF2-α phosphorylation was due to over-expression of the protein, we expressed HSV-1 gD and HIV-1 Vpu and examined for eIF2-α phosphorylation. The expression of gD or Vpu did not result in the phosphorylation of eIF2-α (Additional file 5), indicating that the observed results were likely not due to over-expression. We compared the phosphorylation of eIF2-α by the E protein of SARS-CoV-2 with the E proteins from SARS-CoV, MERS-CoV, and HCoV-OC43 normalization of protein lysates for β-actin. Our results indicate that the expression of each E protein resulted in the phosphorylation of eIF2-α and that the level of phosphorylation of eIF2-α was approximately the same with each E protein (Additional file 5). These results indicate that phosphorylation of eIF2-α did not contribute to the differences in restriction. We also analyzed E-transfected cells for their ability to induce autophagy. HEK293 cells were transfected with each of the four E proteins and at 48 h analyzed for lipidation of LC3-I. Immunoblots of the cell lysates from transfected cells revealed that all four E proteins resulted in LC3-I lipidation to some extent but that LC3-I lipidation did not correlate with the HIV-1 restriction pattern (Additional file 6).

### Expression of SARS-CoV-2 and SARS-CoV E proteins induce higher levels of caspase 3 activity

**Fig. 6:**
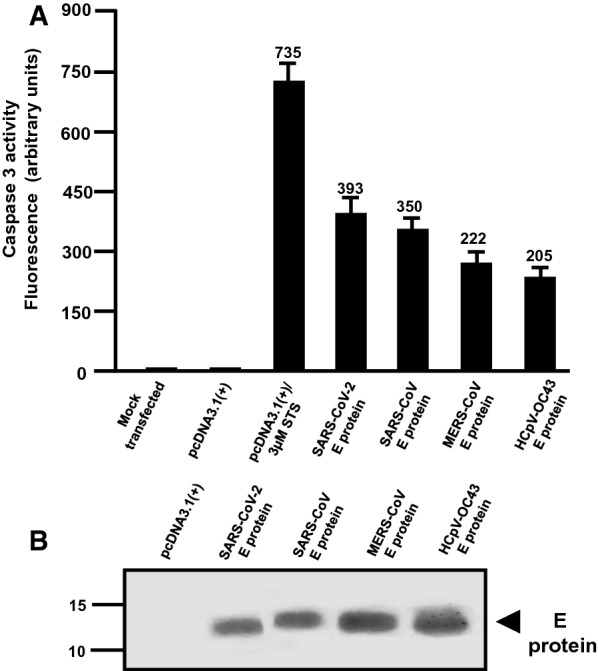
The coronavirus E proteins induce caspase 3 activity. HEK293 cells were either mock-transfected, transfected with pcDNA3.1( +), transfected with pcDNA3.1( +), and treated with 2 µM staurosporine, or transfected with the vectors expressing the E proteins. At 48 h post-transfection, cells were lysed and analyzed for caspase 3 activity according to the manufacturer’s instructions. Fluorescence was measured using a microplate reader using excitation at 342 nm and emission at 441 nm (**A)** and the expression of the E proteins using an anti-HA antibody (α-HA) and immunoblots (**B**). Vectors used to transfect cells are at the top of each lane. Assays were performed at least three times

Previous studies have shown the induction of apoptosis eventually leads to an inhibition of protein synthesis. We determined caspase 3 activity in cells transfected with the empty pcDNA3.1( +) vector, pcDNA3.1( +) plus 2 µM staurosporine (STS) or pcDNA3.1( +) expressing each of the four E proteins. At 48 h post-transfection, cell lysates were prepared and analyzed for caspase 3 activity according to the manufacturer’s instructions. Our results indicate that transfection of HEK293 cells with the empty pcDNA3.1( +) vector plus treatment of cells with STS resulted in significant caspase 3 activity (Fig. 6). Cells transfected with vectors expressing each of the E proteins revealed that the SARS-CoV-2 and SARS-CoV E proteins had the highest level of caspase 3 activity followed by the MERS-CoV and HCoV-OC-43 E proteins. These studies reveal a rough correlation between caspase 3 activity and the levels of infectious HIV-1 production.

### BST-2 down-regulation by E proteins

**Fig. 7:**
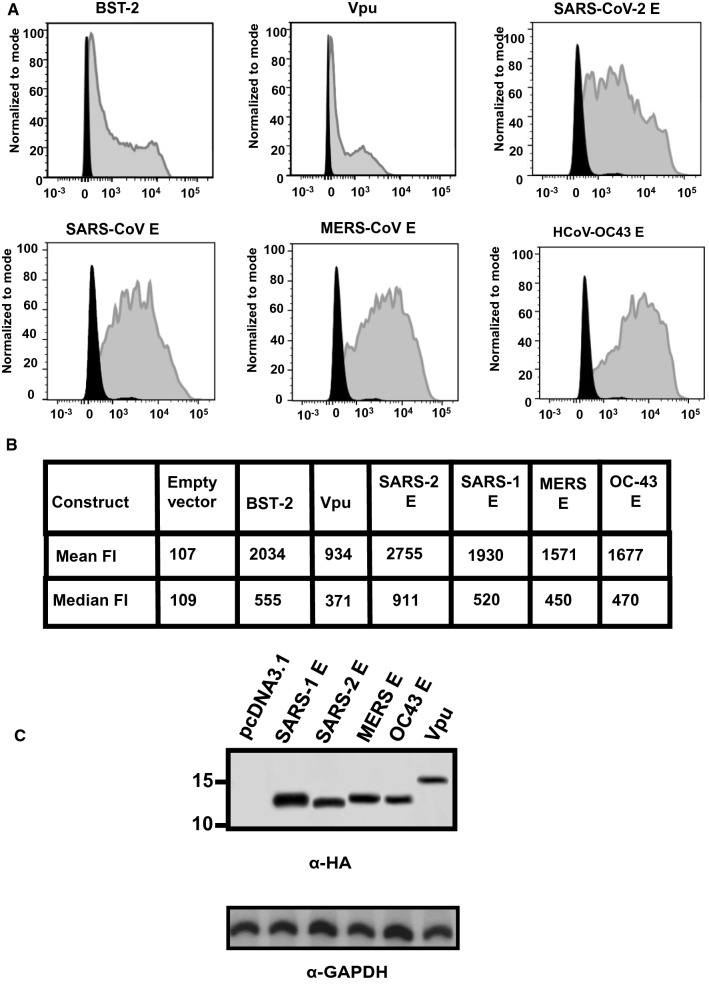
BST-2 down-regulation by Vpu and E proteins. HEK293 cells were co-transfected with either the empty pcDNA3.1( +) vector, the pcDNA3.1( +) vector expressing the HIV-1 Vpu protein, or each of the four E proteins, and a vector expressing human BST-2 protein. **A** At 48 h, the cells were removed by treatment with EDTA/EGTA, stained with a mouse monoclonal antibody directed against BST-2, and subjected to flow cytometric analysis using a BD LSR II flow cytometer. The fluorescent intensities are on the x-axis. **B** Median and mean fluorescent intensities of the BST-2 on the cells from Panel A. **C** Aliquots of cells from the same co-transfection were also analyzed for protein expression by immunoblots using antibodies directed against the HA-tag (E proteins and Vpu) to monitor expression

293 cells were co-transfected with vectors expressing the E proteins or Vpu and the human BST-2 protein. At 48 h, cells were stained for BST-2 using an appropriate anti-BST-2 antibody. The mean fluorescent intensities were calculated using the FlowJo software program (Fig. 7a, b). Our results showed that none of the E proteins down-regulated BST-2 cell surface expression (Fig. 7a, b). However, the HIV-1 Vpu protein significantly down-modulated BST-2 cell surface expression (p > 0.05) (Fig. 7a, b). Analysis of aliquots of cells from the same co-transfections revealed that E proteins and Vpu were all expressed well in co-transfected cells (Fig. 7c).

**Fig. 8:**
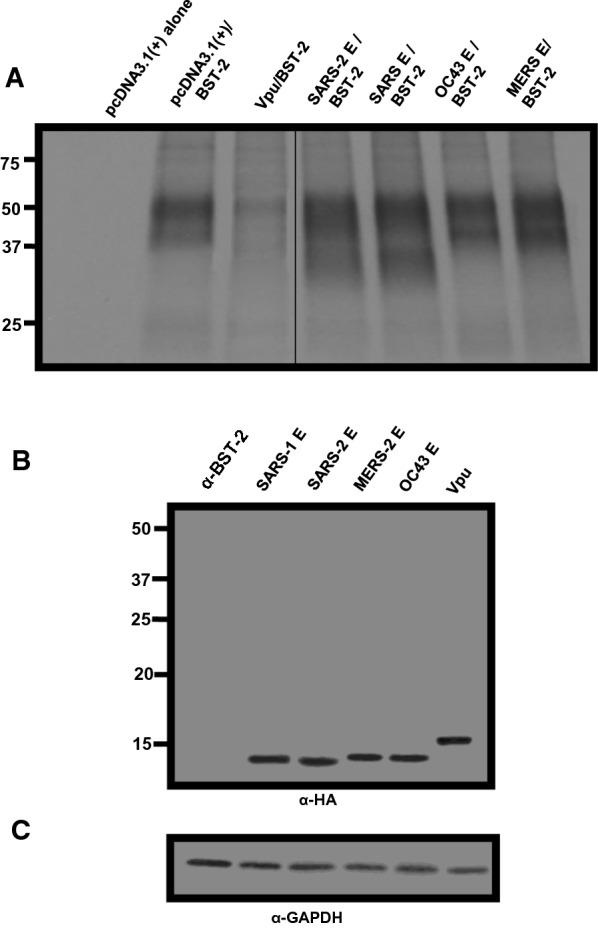
The levels of BST-2 in cells in the presence of β-coronavirus E proteins. HEK293 cells were co-transfected with either the empty pcDNA3.1( +) vector, the pcDNA3.1( +) vector expressing each of the four E proteins, or HIV-1 Vpu and a vector expressing human BST-2 protein. At 30 h post-transfection, cells were starved for methionine/cysteine and radiolabeled with 500 μCi of ^35^S-methionine/cysteine for 16 h. Cell lysates were prepared in RIPA buffer. One-third of the lysate was used to immunoprecipitate BST-2 proteins using an anti-BST-2 antibody (**A**); one-third of the lysate was used to immunoprecipitate E proteins and Vpu (**B**), and one-third of the lysate was used to immunoprecipitate GAPDH to normalize loading (**C**). The line in Panel A is due to the removal of several lanes from the center of the autoradiograph

We next examined the steady-state levels of BST-2 in the presence of the proteins analyzed above. HEK293 cells were co-transfected with vectors as described above and radiolabeled as described in the Materials and Methods section. Cell lysates were prepared, and the BST-2 proteins and E proteins were immunoprecipitated with appropriate antibodies as described in the Materials and Methods section. The results indicate that in the presence of the HIV-1 Vpu, levels of immunoprecipitated BST-2 were significantly less than from cells transfected with the vector expressing BST-2 alone or vectors expressing BST-2 and the E proteins (Fig. 8). Taken together, the E proteins had no effect on BST-2 expression while the Vpu protein caused the down-regulation of BST-2 from the cell surface and a decrease in total amounts of BST-2.

## Discussion

The E protein of coronaviruses is a multi-functional protein that has several roles in the virus replication cycle. It is localized to the ER, ERGIC, and Golgi compartments of the cell and associates with the M protein at the ERGIC during virus maturation [35–37]. Other studies have shown that the E protein of infectious bronchitis virus (IBV) alters the secretory pathway by disrupting Golgi organization leading to the production of larger vesicles capable of transporting virions [38–40]. The role of the E protein in virus maturation varies with the coronavirus. The deletion of the E gene of transmissible gastroenteritis virus (TGEV) completely arrests virus maturation while MHV, SARS-CoV, and HCoV-OC43 expressing non-functional E proteins generate less infectious virus [14, 41–43]. Replacement of the hydrophobic domain of IBV E with that of VSV G protein negatively affected virus maturation [39].

Here, we examined the biological properties of the SARS-CoV-2 E protein in the context of HIV-1. HIV-1 expresses the Vpu protein, which despite having a different sequence, has a similar length, an uncleaved leader sequence, and a similar membrane orientation as coronavirus E proteins. Additionally, both the E protein and Vpu enhance the release of their respective viruses. Several RNA viruses encode proteins known as viroporins, which are virally encoded pore-forming membrane proteins that can alter membranes and ion homeostasis. These proteins are important in entry and/or egress from cells [44–47]. The prototypical viroporin is the M2 protein of influenza A virus and since the identification of its ion channel properties, viroporins have been identified in several different viruses including HIV-1 (Vpu), hepatitis C virus (p7 protein), respiratory syncytial virus (SH protein), classical swine fever virus (p7 protein), and human papillomavirus (E5 protein) [48–55]. The E proteins from SARS-CoV, MERS-CoV, and IBV have been reported to be viroporins while there are no reports of an ion channel activity associated with the HCoV-OC43 E protein [25, 56–62]. Viroporins have been shown to transport different ions but generally are weakly selective for cations (H^+^, K^+^, Na^+^, Ca^++^) over anions and can alter host cell homeostasis at various stages during infection, often involving viral entry and/or release [63]. However, other cellular functions such as apoptosis and vesicular trafficking can also be perturbed by viroporins [64]. Our results revealed the SARS-CoV-2 (and SARS-CoV) E proteins significantly inhibited the unmodified HIV-1 infection while the E proteins from MERS and HCoV-OC43 were less restrictive. As all four E proteins likely have ion channel activity but exhibit differences in infectious HIV-1 production, it likely rules out that viroporin activity is responsible for the observed restriction. However, it is still unknown if differences in the ion selectivity by these different viroporins may have an impact on infectious HIV-1 production. The E proteins of SARS-CoV-2 and SARS-CoV have a high amino acid identity (> 95%) while the E proteins of MERS-CoV and HCoV-OC43 are less related at 37% and 26%, respectively. This suggests that the differences in HIV-1 restriction are likely due to the differences in the amino acid sequences.

Mechanistically, we showed that neither viral integration nor RNA synthesis was impaired by the presence of the SARS-CoV-2 E protein. We observed that in the presence of the SARS-CoV-2 and SARS-CoV E proteins, the levels of HIV-1 protein synthesis were significantly reduced. One possible explanation is that the proteins caused ER stress, which can develop because of Ca ^+  + ^depletion, nutrient deprivation, hypoxia, improperly folded proteins, and point mutations in secreted proteins that stabilize intermediate folding forms or cause aggregation. In response, the cell initiates an unfolded protein response (UPR), which is a cellular pathway that detects and attempts to alleviate ER stress [65–67]. The UPR is initiated by the activation of three canonical ER stress sensors: a) the PKR-like endoplasmic reticulum kinase (PERK); b) activating transcription factor 6 (ATF6), and c) inositol-requiring enzyme 1α (IRE1-α) [68–71]. We showed that expression of all four proteins resulted in the phosphorylation of eIF2-α although the level of eIF2-α phosphorylation did not correlate with the level of infectious HIV-1 produced. We also examined if the expression of the SARS-CoV-2 and SARS-CoV E proteins induced autophagy, which could have led to decreased protein synthesis. Autophagy, first discovered in yeast, is a conserved multi-step degradative process that maintains cell homeostasis by eliminating misfolded/old elements (proteins and organelles) trapped in autophagosomes targeted to fuse with lysosomes to obtain nutrients [72, 73]. Autophagy is also relevant in innate and adaptive immunity against viral infections [74–76]. HIV-1 infection has been shown to induce (macrophages) or inhibit (CD4 + T cells) autophagy [77–79]. During the initial entry step, the HIV-1 gp120/gp41 protein binds the virus to the CD4 receptors and co-receptor CCR5 or CXCR4, initiating an autophagic response in CD4 + T cells. This autophagic process represents an anti-HIV-1 response by the host cell leading to the selective degradation of HIV-1 Tat [80–82]. In the later stages of HIV-1 infection, there is increased activation of the mechanistic target of rapamycin complex 1 (mTORC1) (a regulator of autophagy) which leads to an inhibition of autophagy [82]. Additionally, HIV-1 Nef protein interacts with Beclin 1 and its inhibitor BCL2 [83]. We show here that the expression of each of the four E proteins leads to increased lipidation of microtubule-associated protein 1A/1B-light chain 3 (LC3) to yield LC3-II, which is required for autophagy [84]. However, the pattern of LC3-I lipidation was not consistent with the level of HIV-1 restriction. It is known that the E proteins can induce apoptosis [85–88]. Finally, we analyzed the caspase 3 activity (an effector caspase of apoptosis) in the presence of the four E proteins. Apoptosis can lead to protein synthesis shutdown, which may explain the decrease in HIV-1 protein synthesis in the presence of the E protein. Thus, our results indicate a correlation between caspase 3 activity and HIV-1 restriction.

We also examined the E proteins from SARS-CoV-2, SARS-CoV, MERS-CoV, and HCoV-OC43 for the ability to down-regulate bone marrow stromal antigen-2 (BST-2; also known as CD317 or tetherin). This type II transmembrane protein was first discovered as a cell surface marker for differentiated and neoplastic B cell types [89] and later was shown to be induced by type I and II interferons. It is known that BST-2 activates NF-κB resulting in the activation of IFN-I responses and proinflammatory responses against viruses [90]. The antiviral functions of BST-2 were first demonstrated with HIV-1 which lacked the *vpu* gene [91, 92]. Since its initial identification as an antiviral restriction factor, BST-2 has been shown to tether several enveloped viruses including coronaviruses. BST-2 was shown to inhibit the release of HcoV-229E although the protein responsible for overcoming this restriction factor was not investigated [93]. We showed that, unlike Vpu, the E proteins neither down-regulated BST-2 expression at the cell surface nor caused BST-2 degradation.

In conclusion, we showed that the E protein viroporin from SARS-CoV-2 could not substitute for the Vpu protein but rather the SARS-CoV-2 and SARS-CoV E proteins potently restricted the production of infectious HIV-1 infection. Our data suggest that the expression of the SARS-CoV-2 E protein likely led to apoptosis and inhibition of protein synthesis. These results show for the first time that a viroporin from one virus can inhibit the infection by another virus.

## Materials and methods

### Cells, viruses, and plasmids

HEK293 cells were used for the transfection of vectors expressing coronavirus proteins and the HIV-1 genome. The TZM-bl cell line was used as an indicator to measure HIV-1 infectivity [94–99]. Both cell lines were maintained as previously described [29, 30, 94]. Plasmids with the entire HIV-1 NL4-3 genome (pNL4-3) and pNL4-3Δ*vpu* were obtained from the NIH AIDS Reagent Branch. Plasmids (all pcDNA3.1( +) based) expressing E proteins (SARS-CoV-2 E: accession #QIH45055, SARS-CoV E: accession # AAP13443, MERS-CoV E: accession #ATG84849, and HCoV-OC43 E: accession #ARA15423), and the HIV-1 Vpu protein (strain NL4-3) were synthesized by Synbio Technologies with C-terminal HA-tags and were sequenced to ensure that no deletions or other mutations were introduced during the synthesis. Expression of different coronavirus E proteins was confirmed by transfection with the Turbofect transfection reagent (ThermoFisher) followed by radiolabeling and immunoprecipitation analysis using a mouse monoclonal antibody directed against the HA-tag (Thermo-Fisher, #26183).

### Immunofluorescence studies

To examine the intracellular localization of the SARS-CoV-2 E protein, COS-7 cells grown on 13 mm coverslips were transfected with either the empty pcDNA3.1( +) vector or one expressing the SARS-CoV-2 E protein using Turbofect transfection reagent (ThermoFisher). At 24 h post-transfection, cells were washed in PBS, fixed in 4% paraformaldehyde in PBS for 15 min, permeabilized with 0.2% Triton X-100 in PBS, and blocked for one hour with 22.5 mg/mL glycine and 1% BSA in PBST. The cultures were then incubated at 4C overnight with mouse monoclonal antibody against the HA-tag (Thermo Fisher, #26183) and rabbit polyclonal antibody against ERGIC53 (Proteintech, #13364–1-AP), a rabbit polyclonal antibody against Golgin-97 (Abcam, #ab84340) or a rabbit monoclonal antibody against LAMP-1 (Cell Signaling Technologies #9091). The cells were washed in PBS and incubated with a secondary goat anti-rabbit antibody conjugated to AlexaFluor-488 (Invitrogen, A11008), and a chicken anti-mouse conjugated to AlexaFluor-594 (Invitrogen, A21201) for 1 h. Cells were counterstained with DAPI, and the coverslips were mounted on glass slides with a glycerol-containing mounting medium (Prolong^™^ Diamond Antifade Mountant; Invitrogen). To examine the intracellular localization of the SARS-CoV-2 E protein with the rough endoplasmic reticulum, cis-medial Golgi, or trans Golgi network (TGN), COS-7 cells were co-transfected with the plasmid expressing the E-HA protein and vectors expressing ERMoxGFP (Addgene #68072), mNeonGreen-Giantin (Addgene #98880), or TGN38-GFP (Addgene #128148). Cells were prepared as above and immunostained for E-HA using an anti-HA antibody. Coverslips were viewed with a Leica TCS SP8 Confocal Microscope with a 100X objective and a 2X digital zoom using the Leica Application Suite X (LASX) as previously described. Micrographs of Sects. (0.7 μM) from the center of the cell were photographed and a minimum of 100 cells were examined for each sample, and the results presented in the figures are representative of each sample.

### Analysis of infectious HIV-1 production in the presence of E proteins

To analyze the virus restriction properties of the E proteins, HEK293 cells were transfected with either the empty pcDNA3.1( +) vector, a vector expressing gD (positive control for restriction), gD[ΔTMCT] (negative control for restriction), or E proteins and pNL4-3 [29, 30, 94]. At 48 h post-transfection (pt), the culture medium was collected, clarified by low-speed centrifugation, and the supernatants were analyzed for infectious virus by titration on TZM-bl cells (29, 30, 94–99). All assays were performed at least four times and analyzed for statistical significance using a two-tiered Students *t*-test with cells co-transfected with the empty pcDNA3.1( +) and pNL4-3 set at 100% infectivity.

### Analysis of infectious HSV-1 produced in the presence of SARS-CoV-2 E protein

To determine if the SARS-CoV-2 E protein would restrict the replication of HSV-1, HEK293 cells were transfected with either the empty vector or with a vector expressing SARS-CoV-2 E protein. At 48 h, cells were infected with HSV-1 (0.01 pfu/cell) for 2 h. The cells were collected at 3, 24, and 48 h post-infection, and virus progeny production was determined by titration on Vero cells. Briefly, sterile skim milk was added to the culture, and the cells were scraped into the medium and briefly sonicated. Levels of infectious virus were determined by preparing a series of tenfold dilutions of the culture supernatant followed by inoculation of Vero cells. The number of plaque-forming units was determined by standard procedures.

### Biosynthesis and processing of viral proteins in the presence of E proteins

The biosynthesis and processing of HIV-1 proteins were examined in the presence of the coronavirus E proteins. HEK293 cells were co-transfected with empty pcDNA3.1( +) or the vector expressing E proteins and pNL4-3. At 30 h, the cells were washed and incubated in DMEM without methionine/cysteine for 2 h. The cells were washed and radiolabeled in DMEM containing 500 μCi ^35^S-Translabel (methionine and cysteine, Perkin-Elmer) for 16 h. Cell lysates were prepared, and the culture medium was processed as previously described [29, 30, 94]. HIV-1 proteins were immunoprecipitated using a cocktail of antibodies previously described and are referred to in the figures as anti-HIV antibodies [29, 30, 94]. The E proteins were immunoprecipitated using a monoclonal antibody directed against the HA-tag. Immunoprecipitates were collected by incubation with protein A-Sepharose beads overnight at 4C, the beads were washed with RIPA buffer, and the samples were resuspended in sample-reducing buffer. The samples were boiled, proteins separated by SDS-PAGE (10 or 12.5% gels), and proteins visualized using standard radiographic techniques.

### Analysis of HIV-1 genome integration and viral RNA synthesis

To determine if the E proteins interfered with viral genome integration, we assessed the level of integration by amplification of Alu repeat/*gag* sequences using the previously described procedure [100]. The oligonucleotides used for detecting Alu-gag products were: a) Alu-*gag* forward oligonucleotide primer, MH535: 5'- AACTAGGGAACCCACTGCTTAAG-3'; b) Alu-*gag* reverse oligonucleotide primer: SB704: 5'-TGCTGGGATTACAGGCGTGAG-3'. One µl of sample from the first round PCR and subject to real-time PCR with R/U5 primers (forward primer: M667-5ʹGGCTAACTAGGGAACCCACTGC-3ʹ; reverse primer: AA55 − 5ʹCTGCTAGAGATTTTCCACACTGAC-3ʹ) and probe (HIV FAM-5ʹ- TAGTGTGTGCCCGTCTGTTGTGTGAC-3ʹTAM) [98] with each experiment, a standard curve of the amplicon being measured was run in duplicate ranging from 10 to 1 × 10^11^ copies plus a no-template control. Reactions contained 1 × premix (Takara), 0.5 µM forward primer, 0.5 µM reverse primer, 0.25 µM probe primer, 100–500 ng of template DNA, and 1X RoxDyeII in a 20 µl volume. After initial incubations of 95 °C for 30 s, 40 cycles of amplification were carried out at 5 s at 95 °C, and 34 s at 60 °C. Reactions were analyzed using the ABI Prism 7500 sequence detection system (PE-Applied Biosystems, Foster City, California).

For analysis of the HIV-1 transcription, HEK293 cells were transfected with either the empty pcDNA3.1( +) vector or one expressing the SARS-CoV-2 E. At 24 h post-transfection, cells were infected with pseudotyped HIV-1ΔE/VSV-G at an MOI of 0.1. At 48 h post-transfection, cells were washed, pelleted and the RNA was extracted using the Invitrogen PureLink RNA Extraction kit. The RNA was treated with PureLink DNase I for 15 min at room temperature followed by heat inactivation. The RNA was reverse transcribed using the SuperScript IV RT kit and oligo(dT) 15 primer. The mixtures were treated with RNase H for 30 min at 37 C. Real-time quantitative PCR was performed using primers and procedures previously described: Tat forward, ES2440: 5ʹ-GTCAGCCTAAAACTGCTTGTACCA-3ʹ; Tat reverse, ES2445: 5ʹ-GCCTGTCGGGTCCCCTC-3; and Tat probe, MH603: 5’-(FAM)-CTCCTATGGCAGGAAGA-(TAMRA)-3ʹ. A standard curve of the RNA amplicon was run ranging from 10 to 1 × 10^11^ copies. Reactions containing 1 × premix (Takara), 0.5 μM forward primer, 0.5 μM reverse primer, 0.25 μM probe, and 1 × RoxDyeII in a 20 μl volume. The thermal cycle is 95 C for 30 s, followed by 40 cycles of amplification for 5 s at 95C and 34 s at 60C. Reactions were analyzed using the ABI Prism 7500 sequence detection system (PE-Applied Biosystems, Foster City, California).

### Analysis of the phosphorylation of eIF2-α and LC3-I lipidation

To determine if the expression of the E protein of SARS-CoV-2 resulted in phosphorylation of eIF2-α, HEK293 cells seeded in 6-well plates were either left untransfected or transfected with 1 μg of pUC19 or of a SARS-CoV-2 E-expressing plasmid. The cells were harvested at 48 h pt and equal amounts of proteins were analyzed by western blot for p-eIF2-α or total eIF2-α (both antibodies were obtained from Cell Signaling). Expression of E was detected using an HA-tag antibody (Invitrogen). β-actin was used as a loading control. To determine that phosphorylation of eIF-2α was not an artifact of over-expression, we also transfected cells with vectors expressing the Vpu of HIV-1 and the gD of HSV-1.

For analysis of LC3-I lipidation, cells were solubilized in triple-detergent buffer (50 mM Tris–HCl [pH 8], 150 mM NaCl, 0.1% sodium dodecyl sulfate, 1% Nonidet P-40, 0.5% sodium deoxycholate, 100 μg of phenylmethylsulfonyl fluoride per/ml) supplemented with phosphatase inhibitors (10 mM NaF, 10 mM β-glycerophosphate, 0.1 mM sodium orthovanadate) and protease inhibitor cocktail (Sigma) and briefly sonicated. The protein concentration was determined with the aid of the Bio-Rad protein assay (Bio-Rad Laboratories). Ten to forty micrograms of total proteins per sample were subjected to further analysis. The rabbit polyclonal antibody against LC3-B (Novus Biological) was used in a 1:5,000 dilution.

### Analysis of caspase 3 activity by E proteins

To determine the levels of apoptosis, cells expressing the E proteins were analyzed using the colorimetric Enz-Chek Caspase-3 assay kit according to the manufacturer’s instructions (Molecular Probes, E-13183). HEK293 cells were either mock-transfected, transfected with pcDNA3.1( +), transfected with pcDNA3.1( +), and treated with 2 µM staurosporine, or transfected with the vectors expressing the E proteins. 48 h pt, cells were harvested, lysed, and centrifuged to clear cellular debris. The supernatants were collected and reacted with Z-DEVD-AMC substrate in a microplate for one hour at room temperature. Fluorescence was measured with the BioTek Synergy H1 microplate reader using excitation at 342 nm and emission at 441 nm. Samples were normalized to the total protein used in each sample. Caspase-3 activity in the pcDNA3.1( +) transfected sample was set as the baseline control.

### Analysis of BST-2 down-regulation by various E proteins

We determined if the E proteins from four coronaviruses (SARS-CoV-2, SARS-CoV, MERS-CoV, HCoV-OC43) were capable of down-regulating cell surface bone marrow stromal antigen 2 (BST-2). HEK293 cells were co-transfected with either empty pcDNA3.1( +) vector and pcDNA3.1( +) expressing the human BST-2 protein, or pcDNA3.1( +) expressing each of the proteins described above and BST-2. Cells co-transfected with vectors expressing HIV-1 Vpu and BST-2 was used as a control for BST-2 down-regulation. At 48 h, the cells were stained with a mouse monoclonal antibody directed against BST-2 and subjected to flow cytometric analysis using a BD LSR II flow cytometer. Aliquots of cells from the same co-transfections were also analyzed for protein expression by immunoblots using antibodies directed against the HA-tag (E proteins and Vpu). To analyze if the above proteins led to a steady state reduction in BST-2, HEK293 cells were co-transfected with vectors expressing either four E-HA proteins or HIV-1 Vpu-HA, and a vector expressing human BST-2. At 36 h post-transfection, cells were starved of methionine/cysteine for 2 h and radiolabeled in methionine/ cysteine-free media containing 500 μCi of ^35^S-methionine/cysteine for 16 h. Cells were washed, lysed in 1X RIPA buffer, and clarified by centrifugation. The lysates were transferred to new tubes and BST-2 (using an anti-BST-2 antibody), the E proteins, and Vpu (using an anti-HA antibody) or GAPDH (using an anti-GAPDH antibody; to equalize loading) immunoprecipitated. The immunoprecipitated proteins were washed in RIPA buffer and boiled in the sample-reducing buffer. The proteins were separated using SDS-PAGE and visualized by standard radiographic techniques.

## Supplementary Information

**Additional file 1.** The expression of the SARS-CoV E proteins are restricted to intracellular compartments of the cell

**Additional file 2.** The expression of the SARS-CoV E proteins are restricted to intracellular compartments of the cell

**Additional file 3.** The expression of the SARS-CoV E proteins are restricted to intracellular compartments of the cell

**Additional file 4.** The SARS-CoV-2 E protein did not alter HIV-1 integration or *tat *transcription

**Additional file 5.** The phosphorylation of eIF2-α by E proteins

**Additional file 6.** LC3-I lipidation by the E proteins

## Data Availability

All data generated or analyzed during this study are included in this published article and its additional files.

## Abbreviations

HIV-1: Human immunodeficiency virus type 1
SARS-CoV-2: Severe acute respiratory syndrome coronavirus 2
E: Envelope protein
Vpu: Viral protein unknown
BST-2: Bone marrow stromal factor 2
HSV-1: Herpes simplex virus type 1
