# Supplementary figures and images for: The envelope proteins from SARS-CoV-2 and SARS-CoV potently reduce the infectivity of human immunodeficiency virus type 1 (HIV-1)
Source: Retrovirology. 2022 Nov 19;19:25. doi: 10.1186/s12977-022-00611-6 (PMC9675205; doi:10.1186/s12977-022-00611-6)

## Slide 1
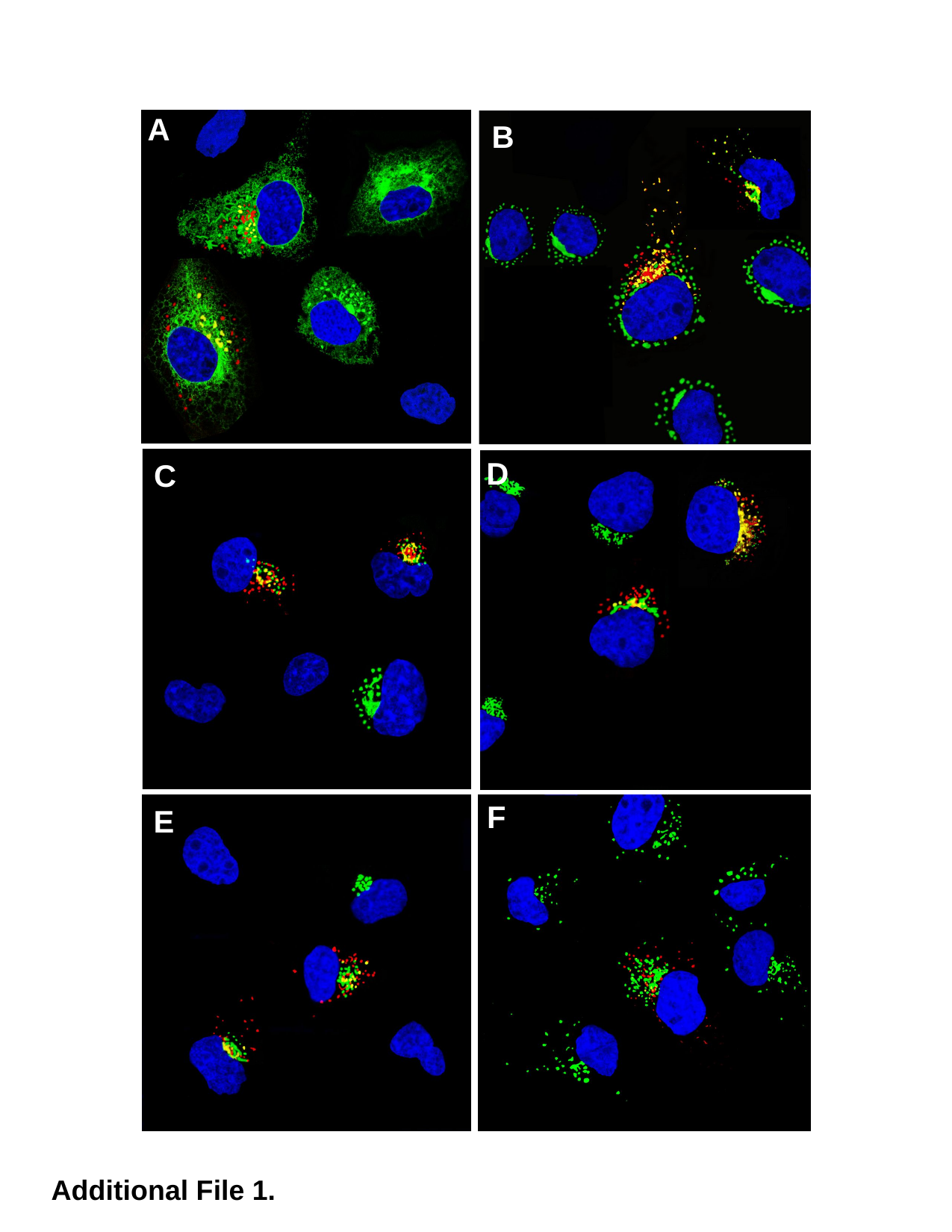

A
B
D
C
F
E
Additional File 1.

Supplement: Supplementary file 1 — Additional file 1. The expression of the SARS-CoV E proteins are restricted to intracellular compartments of the cell [file 12977_2022_611_MOESM1_ESM.pptx]

## Slide 1
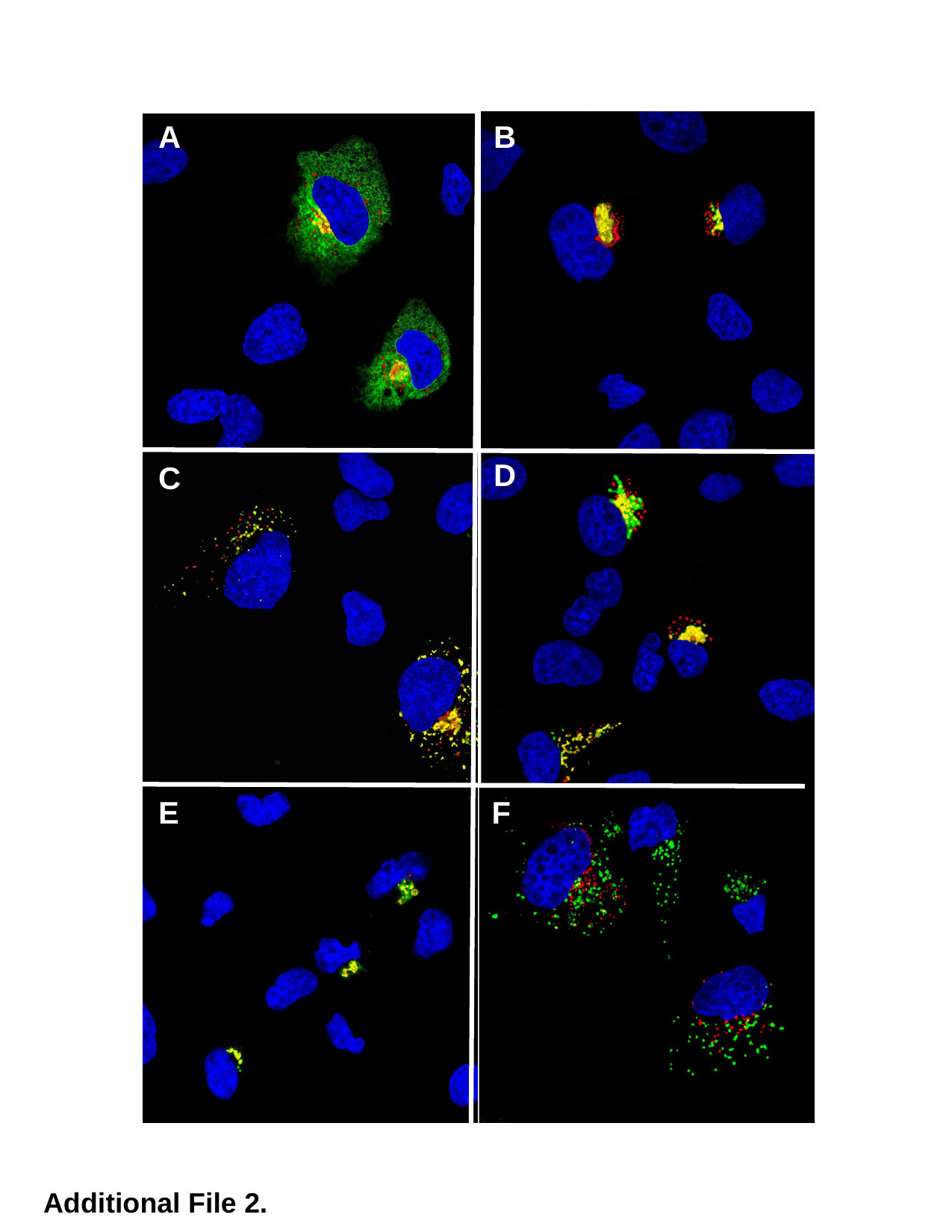

A
B
D
C
E
F
Additional File 2.

Supplement: Supplementary file 2 — Additional file 2. The expression of the SARS-CoV E proteins are restricted to intracellular compartments of the cell [file 12977_2022_611_MOESM2_ESM.pptx]

## Slide 1
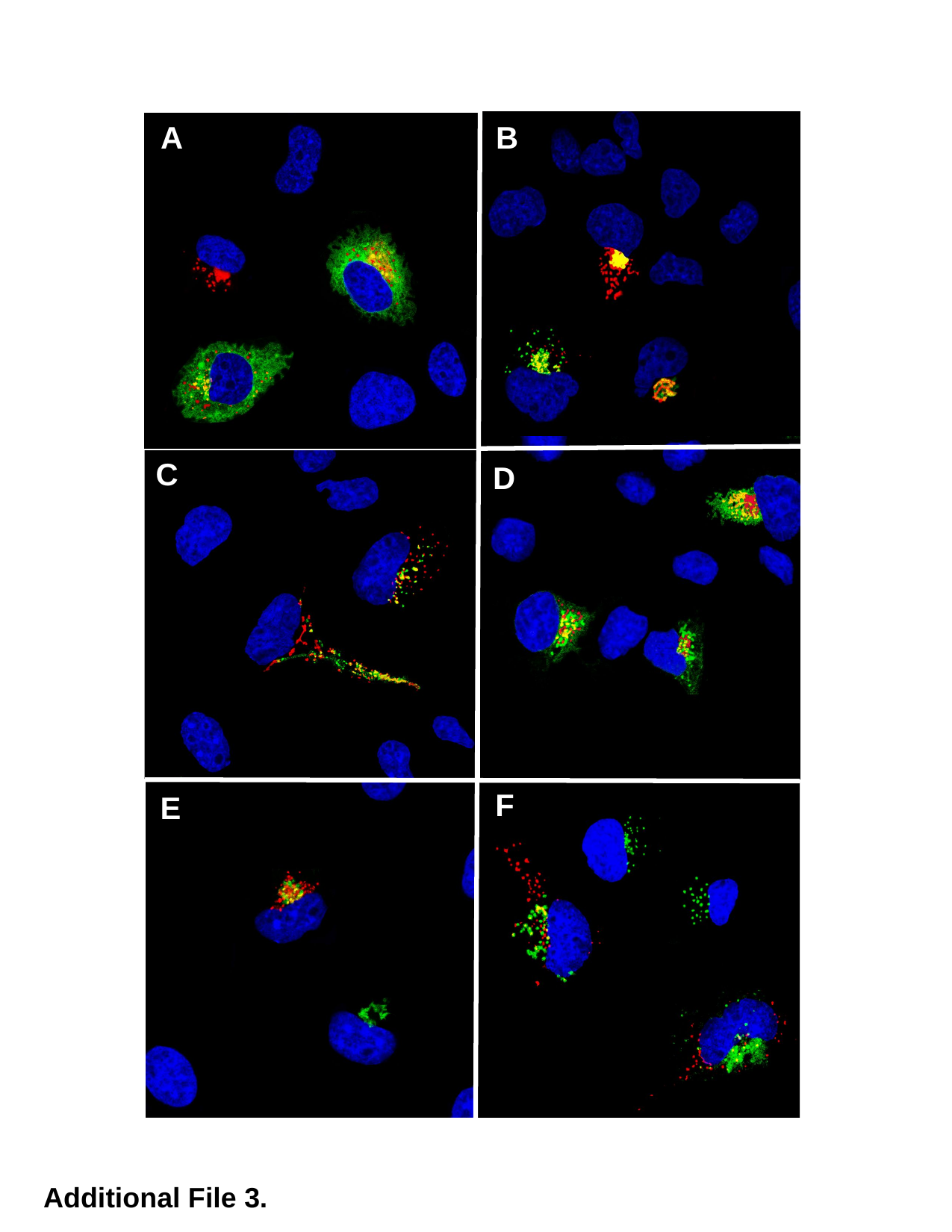

A
B
B
A
D
D
C
E
F
E
Additional File 3.

Supplement: Supplementary file 3 — Additional file 3. The expression of the SARS-CoV E proteins are restricted to intracellular compartments of the cell [file 12977_2022_611_MOESM3_ESM.pptx]
