# Supplementary material for: The envelope proteins from SARS-CoV-2 and SARS-CoV potently reduce the infectivity of human immunodeficiency virus type 1 (HIV-1)
Source: Retrovirology. 2022 Nov 19;19:25. doi: 10.1186/s12977-022-00611-6 (PMC9675205; doi:10.1186/s12977-022-00611-6)
Supplement: Supplementary file 4 — Additional file 4. The SARS-CoV-2 E protein did not alter HIV-1 integration or tat transcription [file 12977_2022_611_MOESM4_ESM.pptx]

## Slide 1
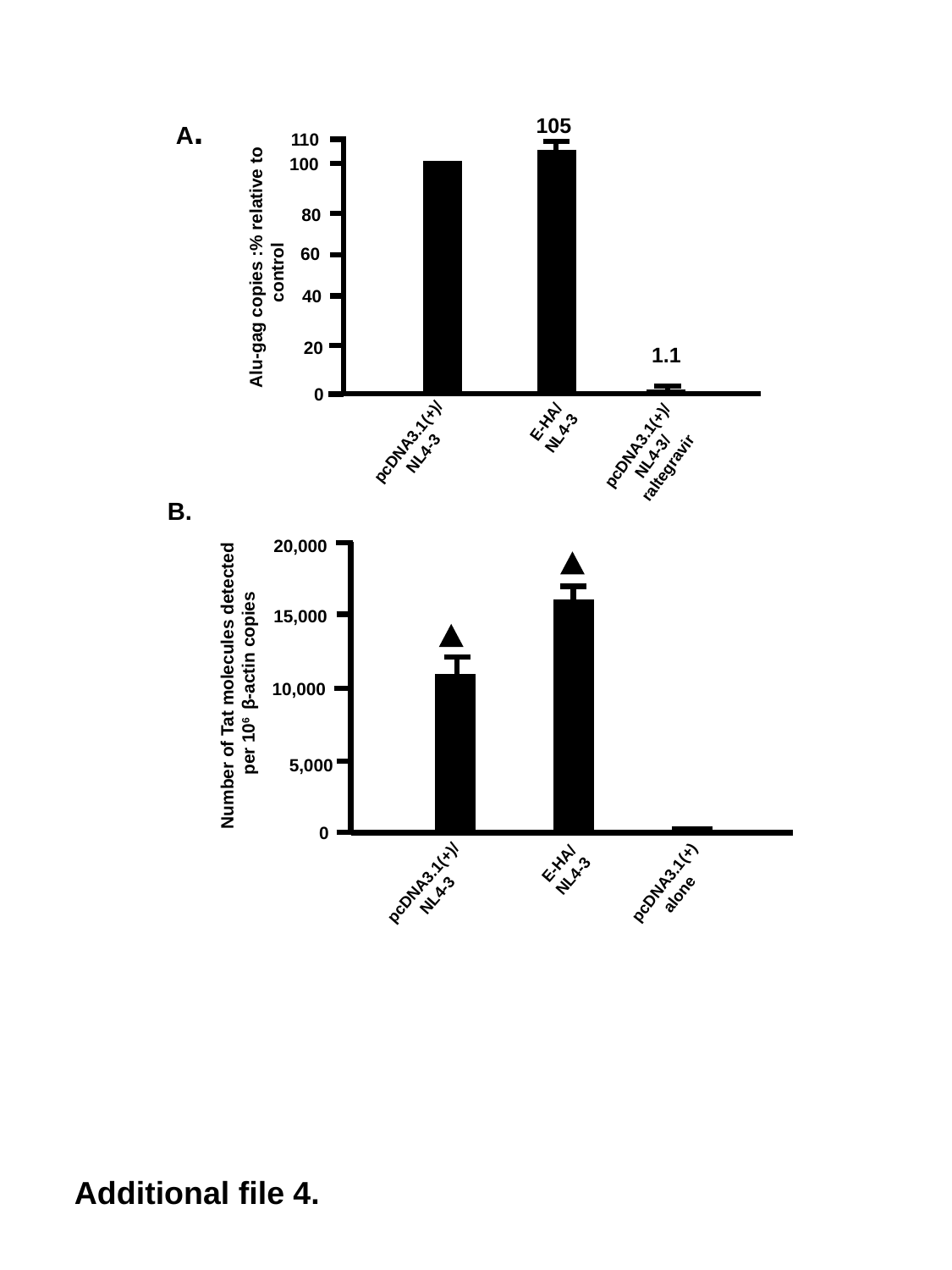

110
100
80
60
40
20
0
E-HA/
NL4-3
pcDNA3.1(+)/
NL4-3
pcDNA3.1(+)/
NL4-3/
raltegravir
Alu-gag copies :% relative to control
1.1
A.
105
20,000
15,000
Number of Tat molecules detected
per 106 β-actin copies
10,000
5,000
0
E-HA/
NL4-3
pcDNA3.1(+)/
NL4-3
pcDNA3.1(+)
alone
B.
Additional file 4.
