# Supplementary material for: The envelope proteins from SARS-CoV-2 and SARS-CoV potently reduce the infectivity of human immunodeficiency virus type 1 (HIV-1)
Source: Retrovirology. 2022 Nov 19;19:25. doi: 10.1186/s12977-022-00611-6 (PMC9675205; doi:10.1186/s12977-022-00611-6)
Supplement: Supplementary file 5 — Additional file 5. The phosphorylation of eIF2-α by E proteins [file 12977_2022_611_MOESM5_ESM.pptx]

## Slide 1
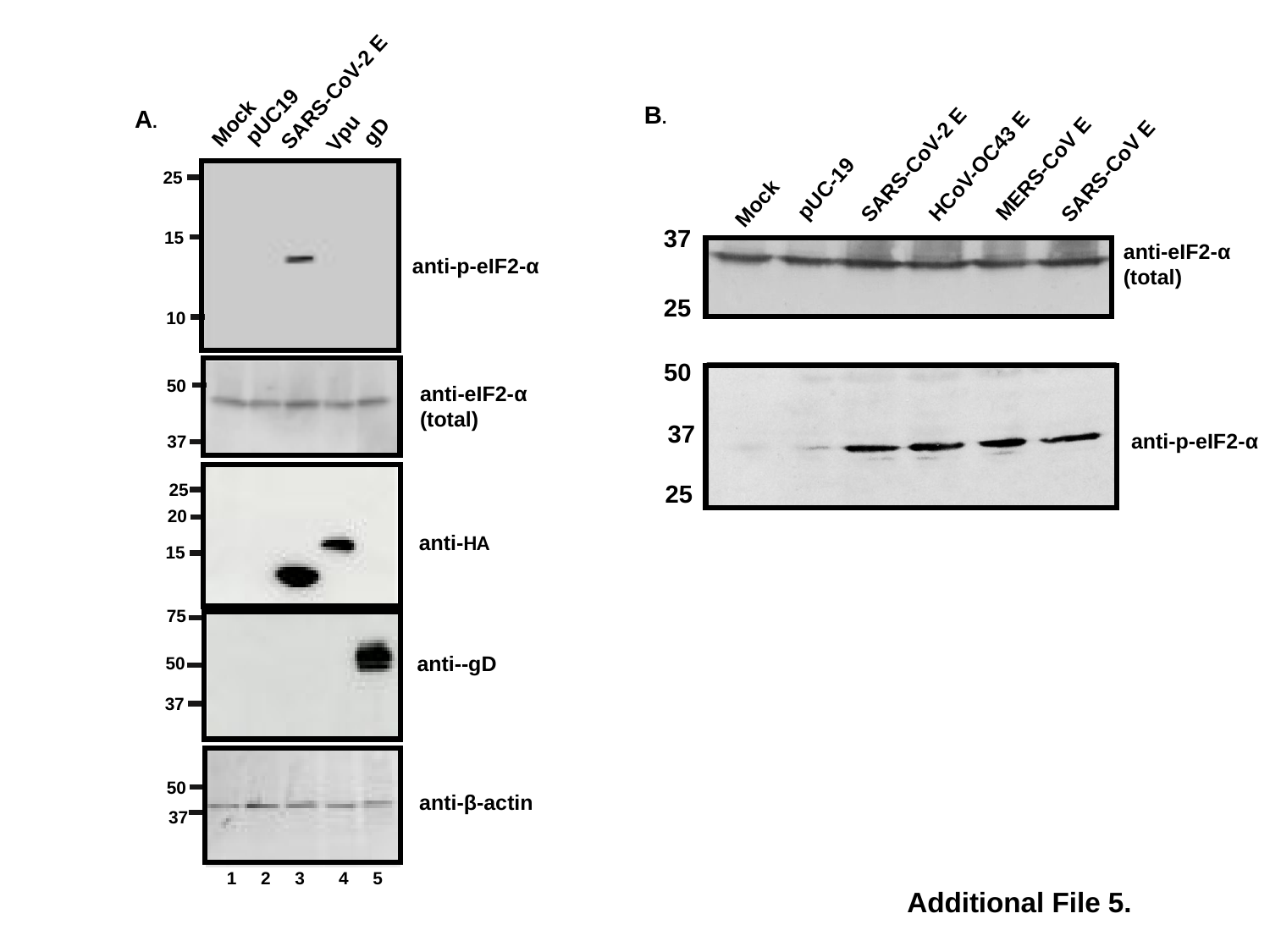

SARS-CoV-2 E
pUC19
gD
Mock
Vpu
B.
A.
25
15
anti-p-eIF2-α
10
50
anti-eIF2-α
(total)
37
25
20
anti-HA
15
75
anti--gD
50
37
50
anti-β-actin
37
1 2 3 4 5
SARS-CoV-2 E
MERS-CoV E
HCoV-OC43 E
SARS-CoV E
pUC-19
Mock
37
anti-eIF2-α
(total)
25
50
37
anti-p-eIF2-α
25
Additional File 5.
