# Supplementary material for: The envelope proteins from SARS-CoV-2 and SARS-CoV potently reduce the infectivity of human immunodeficiency virus type 1 (HIV-1)
Source: Retrovirology. 2022 Nov 19;19:25. doi: 10.1186/s12977-022-00611-6 (PMC9675205; doi:10.1186/s12977-022-00611-6)
Supplement: Supplementary file 6 — Additional file 6. LC3-I lipidation by the E proteins [file 12977_2022_611_MOESM6_ESM.pptx]

## Slide 1
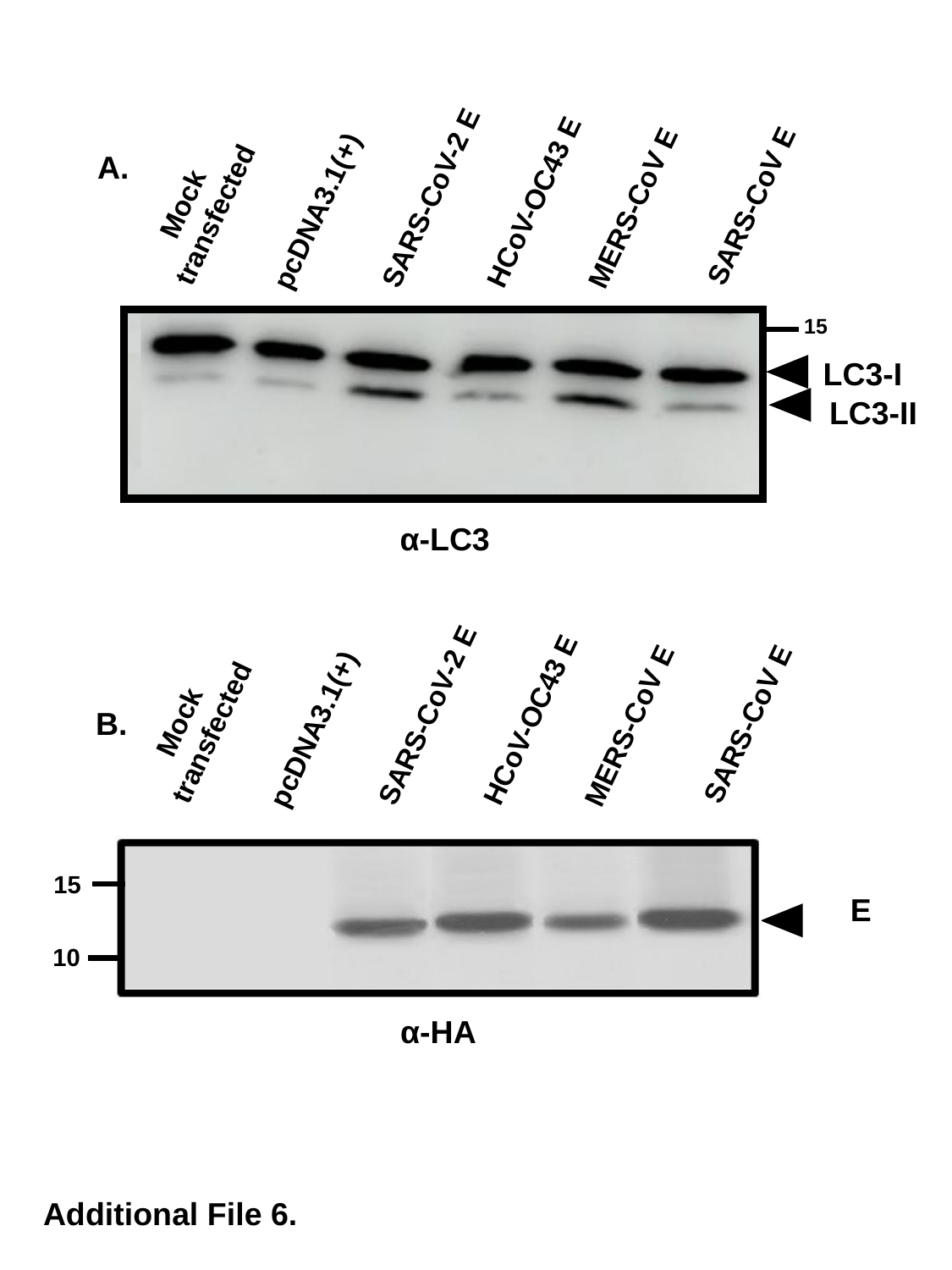

A.
Mock
transfected
SARS-CoV-2 E
HCoV-OC43 E
SARS-CoV E
MERS-CoV E
pcDNA3.1(+)
15
LC3-I
LC3-II
α-LC3
Mock
transfected
SARS-CoV-2 E
HCoV-OC43 E
B.
SARS-CoV E
MERS-CoV E
pcDNA3.1(+)
15
E
10
α-HA
Additional File 6.
